# Hepatitis C Lipo-Viro-Particle from Chronically Infected Patients Interferes with TLR4 Signaling in Dendritic Cell

**DOI:** 10.1371/journal.pone.0000330

**Published:** 2007-03-28

**Authors:** Sophie Agaugué, Laure Perrin-Cocon, Patrice André, Vincent Lotteau

**Affiliations:** 1 Institut National de la Santé et de la Recherche Médicale (INSERM), U851, Lyon, France; 2 Université de Lyon, Lyon-Gerland, France; 3 Hospices Civils de Lyon, Hôpital de la Croix Rousse, Laboratoire de Virologie, France; Centre de Recherche Public-Santé, Luxembourg

## Abstract

**Background:**

Hepatitis C virus (HCV) can be purified from serum of chronically-infected patients in the form of Lipo-Viro-Particles (LVP), which are triglycerid-rich lipoprotein-like particles containing viral RNA and proteins. Since LVP is a constant feature of chronically infected patients, we asked whether purified LVP could interfere with the immune response by acting directly on dendritic cell (DC) function.

**Methods and Findings:**

We have analyzed the impact of LVP on the maturation monocyte-derived DC induced by TLR3 or TLR4 ligands. Following incubation with LVP, immature DC supported weak transient HCV-RNA replication and type I IFN synthesis. This, however, did not lead to viral particle production nor to maturation of DC. LVP-treatment prior to TLR3 stimulation by polyI:C only enhanced the secretion of IL-12, IL-6 and TNFα yielding typical mature DC. In contrast, LVP-treated DC activated by the TLR4 ligand LPS yielded phenotypically mature DC with reduced capacity to secrete both pro- and anti-inflammatory cytokines. Their ability to stimulate allogeneic T lymphocytes was strongly affected since activated T cells produced IL-5 and IL-13 instead of IFNγ. Addition of IFNα prevented the effect of LVP on DC function. Restoration of IFNγ secretion by T cells was obtained by blocking ERK activation in DC, while induction of IL-5 and IL-13 secretion was inhibited by blocking the p38-MAPK pathway in DC.

**Conclusions:**

LVP can interfere with TLR4-triggered maturation of DC, inducing a shift in DC function that stimulates Th2 cells instead of Th1, by a mechanism that is ERK- and p38-MAPK-dependent. The effect of LVP on DC polarization was reversed by IFNα, providing an additional rationale for the interferon therapy of chronically-infected patients. By acting on TLR4 pathway with LVP, HCV may thus exploit a natural protective mechanism of the liver and the intestine normally used to control inflammation and immunity to commensal microorganisms.

## Introduction

HCV infection is a major public health problem because of the high incidence of chronicity which often leads to liver cirrhosis and hepatocellular carcinoma [1]. HCV is a single stranded RNA virus which has remained poorly characterized due to the lack of an efficient cell culture system and purification procedure but the replicon systems now allowing production of viral particles offer promising tools to help understanding the biology of this virus [2]–[4]. The density of plasma HCV-RNA containing particles is surprisingly heterogeneous and the unusually low density of some of these particles suggests an association of the virus with plasma lipoproteins. HCV may simply bind to circulating lipoproteins but an interference occurring during lipoprotein synthesis by infected hepatocytes may also lead to the generation of a hybrid-virus like particle. One form of HCV-RNA containing structures with low density has indeed been purified from plasma of chronically-infected patients [5]–[8]. These particles named Lipo-Viro-Particles (LVP) appeared as large lipoprotein-like structures enriched in triglycerides containing internal viral capsids and carrying viral envelope proteins at their surface [9]. These particles can enter human hepatocytes *in vitro* through the interaction of apolipoprotein B and E with lipoprotein receptors. The presence of LVP is a constant feature of chronicity but their role in HCV infection and disease progression remains elusive. Quasi-species analysis, the presence of HCV proteins in the enterocytes of chronically-infected patients and the biochemical composition of LVP strongly suggest that LVP can originate from both the liver and the intestine [5], [6], [10], [11].

Beside the intriguing question of the generation of such a viral structure, LVP is of particular interest because of their lipoprotein nature and the complex mixture of lipids it can contain. Many immunomodulatory properties have been assigned to native and modified lipids and the immunological properties of native and oxidized lipoproteins are influenced by their content in anti- and pro-inflammatory lipids [12]–[14]. For instance, we and others have shown that oxidative modifications of lipoproteins can be detected by dendritic cells (DC) and lipids generated during oxidation can either induce or inhibit DC maturation [15]–[19]. Many lipids have also been shown to interfere with bacteria LPS and Toll-like receptor (TLR) 4 signaling. The overall data from the literature suggest that natural modification of lipids leads to the formation of both positive and negative regulators of adaptive immune responses.

These immunomodulatory properties of lipids have been exploited by many parasites to subvert and escape the immune system. Lysophosphatidylserine from eggs of Schistosoma mansoni inhibits IL-12 production by DC stimulated with IL-1 and TNFα and induces IL-10-secreting regulatory T cells by a TLR2-dependent process. By contrast, DC treated with the inflammatory cocktail and phosphatidylserine from eggs of the parasite induce IL-4-secreting Th2 cells [20]. High amounts of HETE have been detected in red blood cells parasitized by Plasmodium falciparum, interfering with activation of monocytes and maturation of DC induced by LPS [21], [22]. The excretory-secretory product-62 (ES-62) is a phosphorylcholine-substituted secreted glycoprotein of Acanthocheilonema viteae that exerts immunomodulatory functions via TLR-4. In vitro and in vivo exposure of macrophages, DC and their precursors to ES-62 renders the cells unable to respond normally to LPS, polarizing their differentiation towards a Th2/anti-inflammatory phenotype [23], [24].

There are increasing evidences showing that viruses can interact with TLR signaling. Viral components can either bind to TLR and activate their signaling pathway or block TLR function by interfering with intracellular intermediates. Double-stranded RNA (dsRNA) is generated during viral replication and TLR3 activation can be triggered by the synthetic analog of dsRNA polyinosine-deoxycytidylic acid (poly I:C). Moreover, the HCV serine protease NS3/4A can specifically degrade the adaptor protein TRIF (Toll-interleukin 1 receptor domain–containing adaptor inducing IFNβ) resulting in inhibition of TLR3 signaling in HeLa cells containing replicating HCV RNA [25]. Although TLR4 is well-known for its capacity to detect bacterial lipopolysaccharide (LPS), this receptor can also recognize viral components such as the fusion protein from respiratory syncytial virus (RSV) [26] and the envelope protein of mouse mammary tumor virus (MMTV) [27]. TLR4 is important for clearing RSV infection [28] whereas MMTV maintenance depends on TLR4 stimulation by the virus that induce immunoregulatory IL-10 secretion [29].

We have thus studied the reactivity of DC to LVP and its consequences on cellular maturation induced by different TLR3 and TLR4 ligands. It appeared that LVP induced a TLR4 signaling defect involving ERK and p38-MAPK pathways that can be overcome by alpha interferon (IFNα).

## Materials and Methods

### Patients

Blood samples were obtained from volunteers attending the Liver Unit at Necker Hospital (Paris, France). Blood was collected during a therapeutical bleeding prescribed by Dr. S. Pol (Service d'hepatologie, Hôpital Cochin, Paris). Written informed consent was obtained from each patient in agreement with the institutional review board of the Etablissement Francais du sang and the ethical guidelines of the Declaration of Helsinki. Chronically-infected patients have not been given antiviral therapy for more than six months. Viruses were of genotypes 1a, 1b, 2a, 3 and 4. Similar results were obtained irrespectively of genotype.

### LVP purification

LVP was purified by density gradient ultracentrifugation and immunocapture with microbeads as described [6]. Briefly, the density of plasma (1.0063 g/ml) was raised to 1.055 g/ml using NaBr (Sigma-Aldrich, St Quentin-Fallavier, France). After centrifugation at 100000 rpm, 4 h at 4°C on a TL100 centrifuge (Beckman Instruments, Gagny, France), the low density fraction (density between 1.0063 and 1.055 g/ml corresponding to densities of Very Low Density Lipoprotein (VLDL), Intermediate Density Lipoprotein (IDL) and Low Density Lipoprotein (LDL)) was collected, extensively dialyzed at 4°C against 150 mM NaCl/0.24 mM EDTA pH 7.4 and filtered through 0.22 µm-poresize filters (Millipore S.A., St Quentin, France). Immunoglobulin-coated LVP were then purified by incubating the low density fraction with protein A-coated microbeads (Miltenyi Biotec, Paris, France) and by separation on a magnetic column (Miltenyi Biotec). LVP was collected in Dulbecco's Modified Eagle Medium (DMEM) containing 0.2% BSA (Invitrogen, Cergy Pontoise, France) and stored at 4°C until use.

The light fraction at the same density than LVP (1.0063<d<1.055 g/ml) was purified from plasma of healthy individuals and immunoprecipitated with an anti-apolipoprotein B antibody (Calbiochem, La Jolla, CA) and protein A-microbeads as described above and used as control (IP-LP for immunoprecipitated lipoparticles).

### HCV-RNA quantification

RNA was extracted from 1.5×10^5^ cells using RNeasy mini kit (Qiagen, Courtaboeuf, France), and from LVP and culture supernatants using Nucleospin RNA virus kit (Macherey-Nagel, Hoerdt, France). Known amounts of HCV-RNA were mixed with control cells or supernatants, extracted as samples and used to establish the standard curve. HCV-RNA was then quantified by real-time PCR of the 5′-HCV noncoding region as described [6], [30].

### Monocyte and lymphocyte purification

Human peripheral blood from healthy donors was obtained from the Etablissement Français du Sang (Lyon, France). Mononuclear cells were isolated by density gradient centrifugation using Ficoll-Hypaque, and then centrifuged on a 50% Percoll solution (Amersham Biosciences, Uppsala, Sweden). Two fractions were recovered: monocytes were purified from the light density fraction and T lymphocytes from the high density fraction by immunomagnetic depletion (Dynal Biotech, Oslo, Norway) using a cocktail of mAb: anti-CD19 (4G7 hybridoma; provided by Dr Ron Levy), anti-CD3 (OKT3; American Type Culture Collection, Manassas, VA) and anti-CD56 (NKH1; Beckman Coulter, Fullerton, CA) for monocytes; anti-CD19 (4G7), anti-CD56 (NKH1), anti-CD14 (RMO52), anti-CD16 (3G8) and anti-glycophorin A (11E4B7.6) (all from Beckman Coulter) for T lymphocytes. Monocytes were >90% pure as assessed by CD14 labeling and T lymphocytes>95% pure as assessed by CD3 labeling.

### Generation and treatment of DC

Monocytes (10^6^ cells/ml) were differentiated at 37°C under 5% CO_2 _to immature DC (iDC) in complete RPMI 1640 medium (Invitrogen) supplemented with 10% FCS, 50 ng/ml human recombinant granulocyte-macrophage colony-stimulating factor (GM-CSF) and 62.5 ng/ml human recombinant IL-4 (all from Abcys, Paris, France). For the study on iDC, LVP (4 copies of HCV-RNA/cell) were added at day (d) 5 (for a 48 h-incubation) or at d 6 (for a 24 h-incubation) of differentiation. Control iDC were obtained without any treatment and were CD14^−^ CD1a^+^. Cells and supernatants were collected at d 7. To induce DC maturation, LPS (1 µg/ml; Escherichia coli; Sigma) or polyI:C (2 µg/ml, Amersham Biosciences) were always added at d 6 for 24 h. To analyze the impact of LVP on DC maturation, cells were treated with LVP (4 HCV-RNA copies/cell) either at d 5 (24 h before maturation) or at d 6 (2 h before maturation). In some experiments, IFNα (Intron A, 500 IU/ml; Schering-Plough, Kenilworth, NJ) or IFNβ (500 IU/ml; PBL Biomedical Laboratories, Piscataway, NJ) were added concomitantly to LVP at d 5. When indicated, cells were pre-incubated with an inhibitor of the Mitogen-activated protein kinase/extracellular signal to regulated kinase MEK (40 µM PD98059; Biomol, Plymouth Meeting, PA) or an inhibitor of p38-MAPK (25µM SB203580; Biomol) 30 min before LVP treatment at d 5. Control mature DC (mDC) were obtained at d 7 after addition of 1 µg/ml LPS or 2 µg/ml polyI:C at d 6. All cells and culture supernatants were collected at d 7.

### Phenotype

Phenotype was analyzed by flow cytometry on a FACSCalibur (BD Biosciences, Franklin Lakes, NJ) using FITC-conjugated anti-CD14, -HLA-DR, -CD80, and phycoerythrin (PE)-conjugated anti-CD1a, -CD86, -CD83 and -CD40 (all from Beckman Coulter).

### Endocytosis

Cells were resuspended at 10^6^ cells/ml in complete RPMI/10% FCS medium and were incubated at 37°C for 30 min with 1 mg/ml FITC-T40-dextran (Sigma-Aldrich). Internalization was stopped on ice with cold phosphate-buffered saline (PBS) containing 1% BSA and 0.05% NaN_3_. Cells were washed three times at 4°C in this buffer and the amount of probe internalized was measured by flow cytometry. Control was obtained by incubation of cells for 30 min at 4°C.

### Cytokine assay

Culture supernatants were stored at −80°C until use. Cytokine-specific enzyme-linked immunosorbent assay (ELISA) kits were from Pierce (Rockford, IL) except for IL-12p40 that was from Biosource International (Camarillo, CA).

### Mixed Leukocyte Reaction (MLR)

Primary MLR were conducted in 96-well flat-bottom culture plates. DC recovered at d 7 were extensively washed and resuspended in complete RPMI/10% FCS. These cells were then cocultured with 2×10^5^ allogeneic T cells in a final volume of 200 µl at DC/T cell ratios ranging from 1/10 to 1/40. Culture supernatants were recovered after 2 d of coculture for IL-2 quantification and after 4 d of coculture for the measurement of IL-4, IL-10, IL-5, IL-13 and IFNγ by sandwich ELISA (Pierce).

### Type I IFN detection

Type I IFN was measured by a biological assay as described [31]. Type I IFN level in culture supernatants was quantified by determining the protection of bovine kidney cells against the cytopathic effect of vesicular stomatitis virus. Titers were compared to a standard recombinant IFN (Intron A).

### Immunofluorescence

DC were layered on a microscope slide after cytospin at 500 rpm, 5 min. After fixation by 4% paraformaldehyde and permeabilization by PBS/0.1% triton X100, cells were washed three times in PBS/0.2% BSA and incubated with the primary antibody (biotinylated F(ab′)_2_ fragment from 4F3H2 mAb, bioMérieux SA, Marcy l'Etoile, France) for 30 min. Slides were washed three times with PBS/0.2% BSA and incubated for 30 min with FITC-conjugated streptavidine. After three washes in PBS/0.2% BSA and a final wash in distilled water, slides were mounted in fluorescent mounting medium (Dako, Glostrup, Denmark).

## Results

### Effect of LVP on immature DC

**Figure 1:**
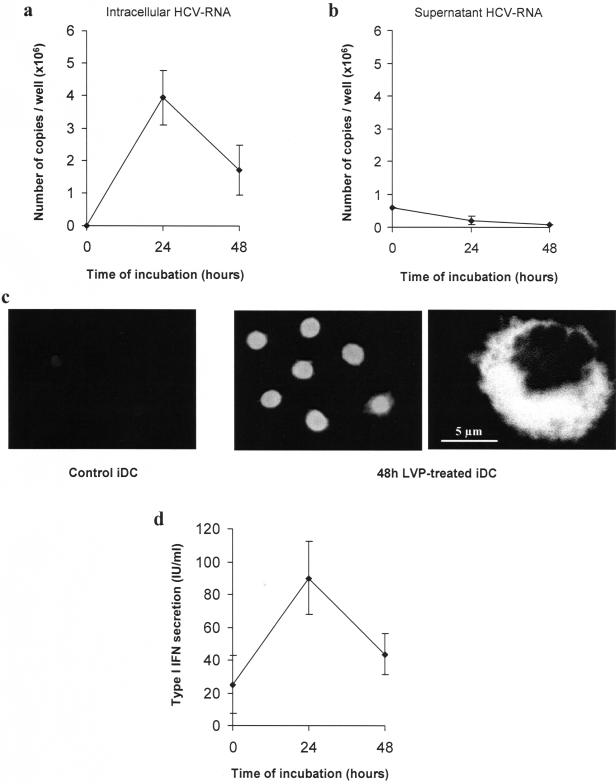
Effect of LVP on immature dendritic cells. (a, b) iDC were incubated for the indicated times with 6×10^5^ HCV-RNA copies (4 HCV-RNA copies/cell). At d 7, HCV-RNA was extracted from cells (a) and supernatants (b) and quantified. Data represent mean copy number/well±sd from triplicates of one representative experiment out of three. (c) NS5A was detected after cytospin of fixed DC (48 h non-treated or LVP-treated DC) using biotinylated 4F3H2 monoclonal antibody and FITC-conjugated streptavidin. Left: 48 h-non treated DC; middle and right: 48 h LVP-treated DC observed in fluorescence microscopy (middle) and in confocal microscopy (right). (d) Type I IFN production was measured by a biological assay in supernatants of non-treated, 24 h or 48 h LVP-treated DC. Titers are expressed as IU/ml with reference to a recombinant IFN. Data represent mean±sd from four independent experiments.

The capacity of LVP to infect iDC was first examined. Lipoproteins can be purified from the plasma by ultracentrifugation on density gradient. In HCV-infected patients, the lipoprotein fraction contains LVP that are low density lipoproteins containing viral capsids. These particles are naturally coated with antibodies allowing their separation from normal lipoproteins by protein A-microbeads. These purified LVP were used throughout this paper. iDC were cultured with 4 HCV-RNA copies/cell for 24 and 48 h before harvesting of supernatants and cells. Quantification of intracellular HCV-RNA was performed by real-time PCR as previously described [6], [30]. Figures 1a and 1b show that LVP entry into iDC can be followed by amplification of HCV-RNA which peaked at 24 h and decreased at 48 h post-infection, suggesting that RNA does not persist in cells. HCV negative strand could also be detected in cells by real time PCR and its identification was confirmed by sequencing (data not shown) [32]. However, detection of HCV negative strand was much less efficient, suggesting that RNA amplification can occur but is not a major event. LVP entry is accompanied by protein synthesis as exemplified by positive NS5A nonstructural protein staining of most DC (figure 1c). Meanwhile, HCV-RNA disappeared from the supernatant indicating that no viral particle was released by infected iDC (figure 1b). LVP induced type I IFN production by DC with a kinetic similar to the RNA quantification curve with a peak at 24 h and a decrease at 48 h (figure 1d).

### LVP does not induce DC maturation

**Figure 2:**
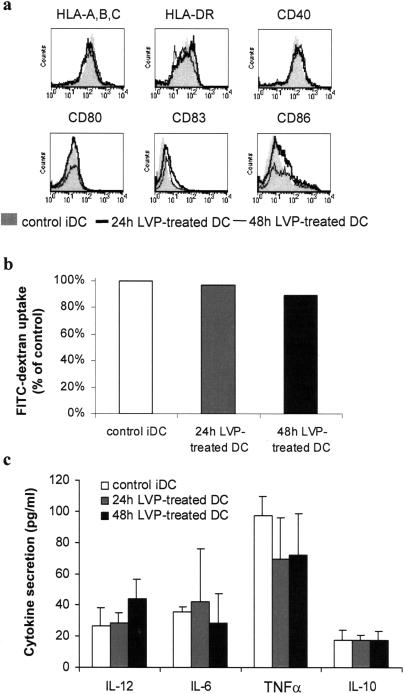
LVP incubation of iDC does not induce maturation. Cells were incubated with LVP as in figure 1. Analysis were performed on non-treated iDC, 24 h and 48 h LVP-treated iDC. (a) Phenotype of non-treated iDC (filled profile), 24 h LVP-treated iDC (thick line) and 48 h LVP-treated iDC (thin line). Representative results of one experiment out of three. (b) Endocytic capacity of non-treated DC (opened bars), 24 h LVP-treated iDC (hatched bars) and 48 h LVP-treated iDC (filled bars). Mean fluorescent intensities were normalized to 100% uptake for non-treated control iDC. Data from one representative experiment out of three. (c) Cytokine secretion in the supernatant of 24 h (hatched bars) and 48 h LVP-treated DC (filled bars) compared to non-treated iDC (opened bars). Data represent mean secretion±sd from four independent experiments.

To investigate the biological functions of LVP, the reactivity of iDC to LVP was analyzed at the phenotypical and functional levels. The phenotype of DC cultured with LVP for 24 h and 48 h was identical to that of control iDC with comparable level of HLA and costimulatory molecules (figure 2a). Endocytic capacity of LVP-incubated iDC was not reduced compared to control iDC and secretion of IL-12, IL-6, TNFα and IL-10 was not significantly modified (figure 2b and c). LVP addition did not affect the viability of DC (data not shown). Thus, incubation of LVP with DC for 24 or 48 h did not induce DC maturation, suggesting that LVP does not provide a maturation signal for DC or that some viral component interferes with the maturation process.

### Influence of LVP infection on DC maturation

**Figure 3:**
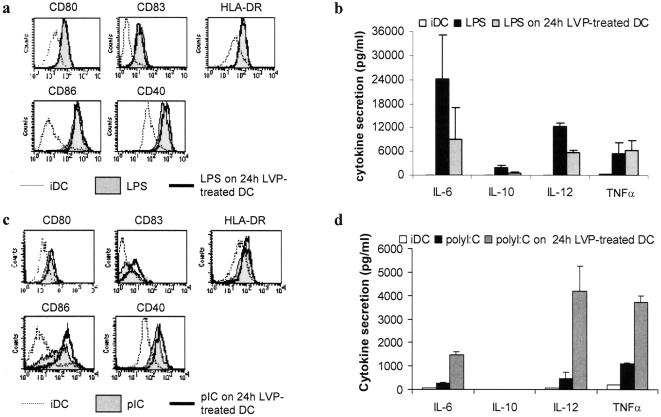
LVP treatment of iDC inhibits maturation induced by LPS but not by polyI:C. Control mature DC (“LPS” or “polyI:C”) were obtained at d 7 after addition of the maturation stimulus at d 6. Control iDC (“iDC”) were obtained at d 7 without any addition. LVP was added to iDC at d 5 and LPS or polyI:C at d 6 for 24 h (“LPS on 24 h LVP-treated DC” or “polyI:C on 24 h LVP-treated DC”). Phenotype and cytokine secretion was analyzed as in figure 2. (a, c) Phenotypic maturation of LVP-treated DC induced by LPS (a) or polyI:C (c). Profiles of control iDC (dotted line), control mature DC (filled profile), and DC treated with LVP 24 h before addition of the maturation agent (thick line). Data from one representative experiment out of three. (b, d) Secretions of cytokines obtained after LPS addition (b) or polyI:C addition (d). Mean secretion±sd of three independent experiments.

A number of viruses have been reported to impair DC maturation [33]. We thus investigated the effect of LVP on the maturation induced by LPS or polyI:C to determine whether LVP could interfere with the maturation process. LPS was used as a TLR4 ligand whereas polyI:C was used as a prototype of TLR3 activation. iDC were treated with LPS or polyI:C at d 6 of differentiation and LVP were added 24 h before the maturation stimulus. Phenotypic and functional analyses were performed 24 h after the induction of maturation. Figure 3a shows that LPS-induced upregulation of HLA-DR and costimulation molecules was not affected by the presence of LVP. All cells were CD14^−^ CD1a^+^ (data not shown). In contrast, DC which were treated with LVP 24 h before LPS addition showed a decreased secretion of IL-12 (50%), IL-6 (50%) and IL-10 (65%) while TNFα secretion was maintained (figure 3b).

LVP-treated DC stimulated by polyI:C showed slight increase of expression of CD83 and CD86 compared to polyI:C-activated DC, while the expressions of HLA-DR, CD80 and CD40 were comparable (figure 3c). LVP-treated DC stimulated by polyI:C showed increased secretions of IL-6, IL-12 and TNFα whereas no IL-10 was secreted. Nevertheless, the levels of secretion were lower compared to those induced by LPS (figure 3d).

Thus these data suggest that LVP interferes with the TLR4 and TLR3 signaling pathways involved in cytokine secretion, resulting in different profiles of production. TLR4 stimulation is impaired while TLR3 pathway seems hyperreactive.

### LVP interferes with TLR4 signaling to induce a Th2-oriented response

**Figure 4:**
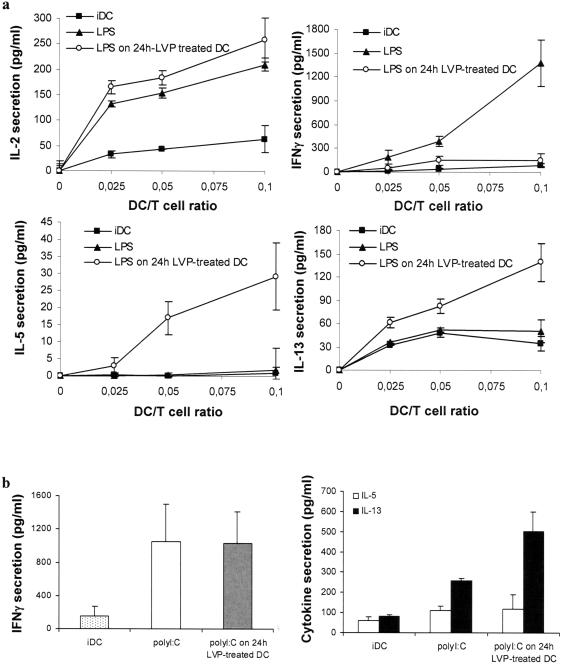
LVP interferes with TLR4 but not with TLR3 signaling to induce a Th2-type response. (a) LVP interferes with T responses elicited by DC stimulated by a TLR4 ligand. Control iDC (▪) were obtained at d 7 without addition of LPS. Control mature DC (▴) were obtained at d 7 after addition of LPS at d 6. LVP was added to iDC at d 5 and LPS at d 6 for 24 h (○). DC recovered at d 7 were washed, resuspended in RPMI/10% FCS and then cocultured with T lymphocytes at DC/T cell ratios ranging from 1/10 to 1/40. IL-2, IFNγ, IL-5 and IL-13 were measured by ELISA in supernatants of co-culture. Data represent means±sd of triplicates of one experiment out of eight. (b) LVP does not interfere with TLR3 signaling in DC. Control iDC (“iDC”) were obtained at d 7 without addition of polyI:C. Control mature DC (“polyI:C”) were obtained at d 7 after addition of polyI:C at d 6. LVP was added to iDC at d 5 and polyI:C at d 6 for 24 h (“polyI:C on 24 h LVP-treated DC”). DC recovered at d 7 were washed, resuspended in RPMI/10% FCS and then cocultured with T lymphocytes at DC/T cell ratios ranging from 1/10 to 1/40. IFNγ, IL-5 and IL-13 were measured by ELISA in supernatants of co-culture. Data are shown for 1/40 DC/T cell ratio. Data represent means ± sd of triplicates of one experiment out of four.

LVP-treated DC matured with LPS or polyI:C were further tested for their ability to stimulate an allogeneic T cell response in MLR experiments. 24 h LVP-treated DC matured with LPS are able to stimulate IL-2 secretion by allogeneic T cells similarly to control mDC. However, in addition to a decreased cytokine production, LPS addition on 24 h LVP-treated DC yielded cells that failed to induce IFNγ production by allogeneic T cells (figure 4a). Although HCV-RNA and protein are present during the early steps of the MLR corresponding to 48 h LVP-treated DC (figure 1a, b), direct evidence of infection progressively disappears during the 4 days of DC/T cell coculture (data not shown). Since Th2 responses have been observed in some HCV-chronically infected individuals and to further evaluate the functional potential of LVP-treated DC, we analyzed the level of various cytokines in MLR supernatants. No IL-4 or IL-10 could be detected in cocultures of T cells and LVP-infected DC treated with LPS (data not shown). In contrast, increased IL-5 and IL-13 levels were detected in the supernatants of coculture of T cells and DC treated with LVP 24 h before LPS (figure 4a). Thus, DC incubated with LVP prior to LPS treatment stimulate T cells to secrete IL-5 and IL-13 and low amounts of IFNγ whereas control LPS-treated DC activate T cells to secrete IFNγ with low amounts of IL-5 and IL-13.

In contrast to what is observed after LPS activation of LVP-treated DC, treatment of DC with LVP before polyI:C activation did not inhibit the production of IFNγ by T cells, therefore correlating to the pro-inflammatory cytokines secreted by these DC (figure 3d, 4b). IL-13 production was still slightly increased but not IL-5 (figure 4b). Neither IL-4 nor IL-10 could be detected in the same supernatants (data not shown).

These data suggest that LVP interferes with the TLR4 signaling to induce a Th2 shift in the T cell response, whereas TLR3 activated DC still induce a Th1 response.

### Purified native lipoproteins do not induce a Th2-biased maturation following TLR4 stimulation

**Figure 5:**
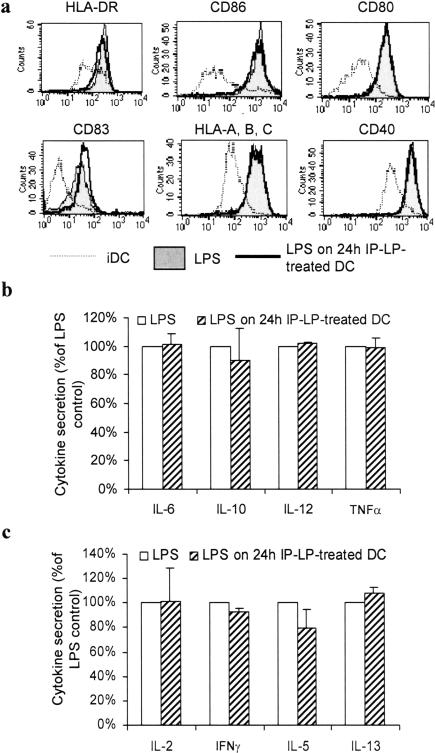
Purified native lipoproteins do not interfere with DC maturation. Control lipoprotein fraction (IP-LP) with the density of LVP was isolated from the blood of non-infected donors by ultracentrifugation and immunoprecipitation with an anti-apolipoprotein B antibody and protein A-microbeads. IP-LP were added to iDC at d 5 and LPS at d 6 for 24 h (“LPS on 24 h IP-LP-treated DC”). (a) Phenotypic maturation of IP-LP-treated DC induced by LPS. Profiles of control iDC (dotted line), control LPS-treated DC (filled profile), DC treated with IP-LP 24 h before LPS treatment (thick line). Data from one representative experiment out of three. (b) Cytokine secretion of LPS-treated DC (opened bars) and DC treated with IP-LP 24 h before LPS treatment (hatched bars). Mean secretion±sd of three independent experiments. Secretion was normalized to 100% for control LPS-treated DC. (c) MLR were conducted as described above with LPS-treated DC (opened bars) and 24 h IP-LP-treated DC matured with LPS (hatched bars). IL-2, IFNγ, IL-5 and IL-13 were measured by ELISA in supernatants of co-culture. Secretion of cytokine was normalized to 100% for control LPS-treated DC. Data are shown for 1/10 DC/T cell ratio.

Although there is no equivalent of LVP particle in healthy donors, we tested the impact lipoproteins prepared as similarly as possible as LVP. They were prepared from the light fraction of lipoproteins from healthy individuals coated with anti-apolipoprotein B antibodies and purified by magnetic positive selection with protein A-coated microbeads (see materials and methods section). The effect of these non-infected immunoprecipitated lipoproteins named IP-LP (for immunoprecipitated lipoparticles) was analyzed on maturation of DC induced by LPS as for LVP (figure 5). Control IP-LP did not inhibit the induction of a mature phenotype (figure 5a) and did not affect cytokine production induced by LPS (figure 5b). In contrast to LVP, control IP-LP did not inhibit the ability of LPS-treated DC to induce IFNγ production and did not increase their potential to stimulate IL-5 and IL-13 secretion by allogeneic T cells (figure 5c).

### IFNα can reverse the effect of LVP on DC polarization

**Figure 6:**
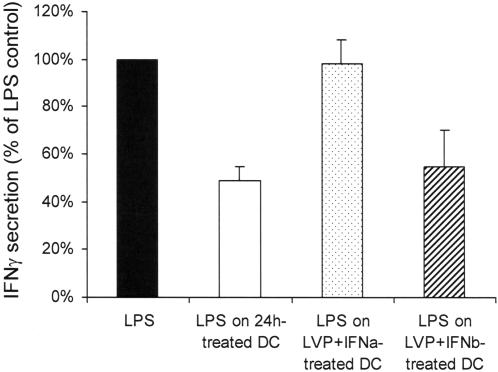
IFNα can restore the ability of LVP-treated DC to induce IFNγ. DC were treated at d 5 with LVP and IFNα or IFNβ, and at d 6 with LPS. MLR were then conducted as described in figure 4 with control LPS-matured DC, 24 h LVP-treated DC matured with LPS, DC treated with IFNα and LVP 24 h before LPS addition, and DC treated with IFNβ and LVP 24 h before LPS addition. IFNγ was measured in supernatants of cocultures by ELISA and normalized to 100% for control LPS-treated DC.

IFNα is an anti-viral cytokine that is currently given to HCV chronically-infected patients with a certain level of success. We thus treated DC with IFNα and LVP to simulate an *in vitro* therapy. IFNα was added at d 5 concomitantly with LVP, and LPS was added at d 6. IFNβ was used as control. These DC were then functionally tested in MLR experiments. As shown in figure 6, addition of IFNα reversed the action of LVP, and DC incubated with IFNα and LVP could mature normally after TLR4 engagement and stimulate the production of IFNγ by T cells. IFNβ which is not able to block viral replication did not reverse the action of LVP on maturation of DC induced by TLR4 stimulation.

### MEK and p38-MAPK are involved in the modification of DC polarization

**Figure 7:**
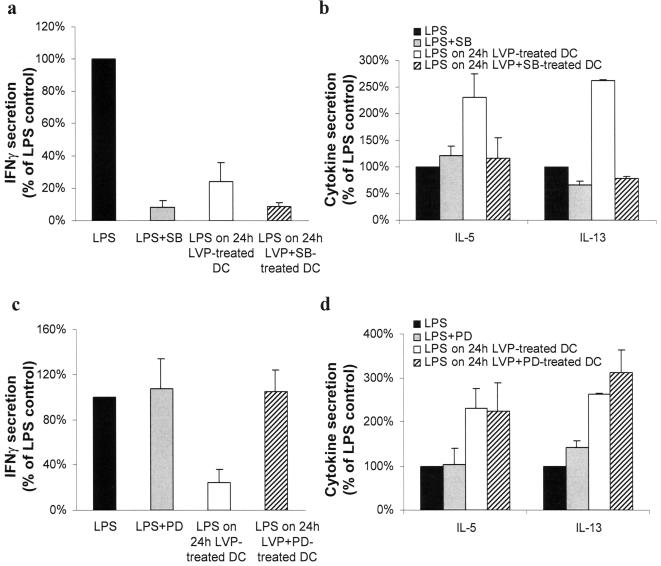
MEK and p38-MAPK pathways are involved in DC polarization. (a, b) MLR were conducted as described in figure 4 with control LPS-matured DC (filled bars), LPS-matured DC pre-treated with SB203580 (SB) (grey bars), 24 h LVP-treated DC matured with LPS (opened bars), DC pre-treated with SB before LVP incubation and LPS treatment (hatched bars). Secretion of cytokine was normalized to 100% for control LPS-treated DC. IFNγ (a) and IL-5 and IL-13 (b) were measured by ELISA in supernatants of co-culture. Mean±sd are shown. Secretions are in the range of 260–2600 pg/ml for IFNγ, 20–80 pg/ml for IL-5 and 150–370 pg/ml for IL-13. (c, d) MLR were conducted as described in figure 4 with control LPS-matured DC (filled bars), LPS-matured DC pre-treated with PD98059 (PD) (grey bars), 24 h LVP-treated DC matured with LPS (opened bars), DC pre-treated with PD before LVP incubation and LPS treatment (hatched bars). Secretion of cytokine was normalized to 100% for control LPS-treated DC. IFNγ (c) and IL-5 and IL-13 (d) were measured by ELISA in supernatants of co-culture.

Many kinases have been shown to play a role in TLR4 maturation of DC and in the type of T responses they elicit. p38-MAPK is mainly involved in CD83 induction, CD80 and CD86 upregulation and in TNFα and IL-12 secretions [34], [35]. ERK has also been shown to be phosphorylated upon TLR4 signaling, but its involvement in TLR4 maturation is still controversial, depending on the culture system [34], [36], [37]. We thus examined the effect of different kinase inhibitors on the ability of DC to stimulate allogeneic T cells. SB203580 is a specific inhibitor of p38-MAPK and PD98059 inhibits MEK activation and prevents ERK phosphorylation. The inhibitors are added at d 5, 30 minutes before LVP. Non-infected DC treated with the inhibitor of p38-MAPK before TLR stimulation were not able to stimulate IFNγ secretion by T lymphocytes. Therefore it was not possible to correlate the action of LVP on DC following LPS stimulation (absence of induction of IFNγ secretion by T cells) to the activation of this pathway (figure 7a). In contrast, blocking p38-MAPK pathway in non-infected DC has no significant effect on IL-5 and IL-13 secretion by T cells. Blocking p38-MAPK pathway in LVP-treated DC yielded cells that lost their ability to stimulate IL-5 and IL-13 secretion by T cells (figure 7b). Moreover, blocking MEK-ERK pathway in DC before LVP incubation and LPS treatment yielded cells with a restored ability to stimulate IFNγ secretion by T cells without affecting IL-5 and IL-13 production (figure 7c, d). These data strongly suggest that LVP could influence DC polarization following TLR4 stimulation by interfering with both p38-MAPK and MEK/ERK pathways.

## Discussion

Several groups have reported that DC from chronically infected patients may have impaired function in vivo and reduced maturation capacity ex vivo but this has been challenged by others who reported that DC from patients could normally function [38]–[46]. Although differences in the experimental procedures could explain this discrepancy, it is likely that DC remain globally functional during the infection because the patients appear immunologically competent. Rather, a precise function of DC could be targeted during the infection that would specifically impair HCV clearance without affecting global immunity. In this study, purified LVP were used to demonstrate that HCV clinical isolate can interfere with the function of DC from non-infected donors in a discrete manner. LVP interfere with the TLR4 pathway to generate mature DC that fail to stimulate production of IFNγ by T cells while that of IL-5 and IL-13 was induced. This Th2 bias observed in these specific conditions is not in favor of efficient HCV-specific CTL responses and could favor chronic infection [47]–[49]. This process can be reversed by IFNα. Although the precise mechanism for TLR4 pathway interference by LVP has not yet been identified, the data show that the engagement of the MEK-ERK and p38-MAPK pathways is involved in the inhibition of IFNγ production and stimulation of IL-5 and IL-13 synthesis, respectively. This is consistent with previous studies showing the relative contribution of these two pathways in DC polarization [35], [37], [50].

LVP entry into DC leads to incomplete infection cycle that ends after a transient RNA replication and protein synthesis. LVP induce various amounts of IFNβ production by DC but IFNα was never detected. We do not have the molecular explanation for the lack of IFNα production but it is known that monocyte-derived DC are weak producers of IFNα in response to virus or TLR stimulation while they produce substantial amounts of IFNβ, probably because the express low levels of interferon regulatory factor 7 (IRF-7) [51]. Several groups have reported a default in IFNα production in chronically-infected patients essentially by looking at the frequency and functionality of plasmacytoid DC [43]–[45], [52], [53]. The effect of LVP on DC was reversed by IFNα in vitro but to what extend IFNα therapy can prevent the effect of LVP on DC in vivo remains to be determined.

Infectious agents have evolved strategies that disrupt signaling pathways normally leading to a protective cellular reaction. TLR3 signaling is not significantly disrupted by LVP and DC appear even more reactive to the TLR3 ligand polyI:C, leading to increased secretion of cytokines. This is an additional indication that LVP do not really infect DC stricto sensus and that the HCV NS3/4A protease is not expressed at a level allowing an efficient cleavage of the TLR3 adaptor protein TRIF that would inhibit TLR3 signaling [25]. Interestingly, the triggering of TLR3 by dsRNA can result in attenuation of the TLR4 pathway [54]. The stimulation of TLR3 by HCV dsRNA could theoretically occur and interfere with the TLR4 pathway but is very unlikely regarding the weakness of HCV-RNA replication in DC. Direct interference with TLR4 signaling may impair anti-viral innate reaction of an infected cell. Interference with TLR2 signaling has already been reported with HCV protein core in human monocytes [55]. Although no binding of an HCV protein to TLR4 has yet been reported, this possibility cannot be excluded.

Lipid remodeling occurs during HCV infection but has not been studied from an immunological point of view. One aspect of this remodeling is the presence of LVP in the blood and infected organs of chronically-infected patients. Although the precise biochemical composition of LVP has not yet been elucidated, we and others have described the lipoprotein-like structure of these particles and their enrichment in triglycerides [5]–[9]. Because modified endogenous or exogenous lipids are detected by DC and can interfere with TLR4 signaling, one intriguing possibility is that LVP could interfere directly with the TLR4 pathway via some of its lipid components [23], [24], [56]–[58]. Alternatively, LVP could interfere with TLR4 signaling by modifying the lipid composition of the plasma membrane of DC similarly to what has been described for polyunsaturated fatty acids that inhibit the induction of Th1 responses possibly by insertion in the membrane [59]. The identification of bioactive lipids that could be involved in the effect of LVP on DC is in progress but is a complex task because of the likely possibility that DC may react globally to LVP by integrating different quality signals delivered by various categories of lipids. Our preliminary lipidomic analysis confirmed the lipoprotein-like structure of LVP with some features not found in normal low density lipoproteins.

A growing list of apparently unrelated molecules can interfere with TLR4 signaling. Among those are microbial products from bacteria, viruses, and parasites. This underlines the crucial role of TLR4 pathway in the innate recognition of pathogens but may also reveal microbial strategies evolved to facilitate infection. Physiological relevance of TLR4 signaling interference by LVP may relate to the origin of these particles and their biochemical composition. The liver lies directly downstream of the gut and is therefore constantly exposed to antigens and microbial products derived from the intestine. Control of liver inflammation and tolerance to gut-derived antigens (food antigens and commensal bacteria) are likely to rely, at least in part, on functional characteristics of hepatic and intestinal antigen presenting cells. One of these characteristics is the modulation of TLR4 signaling under the dual pressure of protecting the host from pathogenic infections and coexistence with the myriad commensal organisms. Many mechanisms are involved to limit TLR4 responses to these commensal bacteria: decreased expression of TLR4, decreased IL-1 receptor-activated kinase (IRAK) activity, increased expression of molecules involved in TLR4 negative signaling (Toll-interacting protein (Tollip), single immunoglobulin IL-1-related receptor (SIGIRR), peroxisome proliferator-activated receptors γ (PPARγ) [60]. Several lines of evidence suggest that the intestine and more specifically enterocytes can be infected by HCV [11] and that LVP could derive from both the liver and the intestine [9]. By acting on TLR4 pathway with LVP, HCV may thus exploit a natural protective mechanism of the liver and the intestine normally used to control inflammation and immunity to commensal microorganisms. Accordingly, intestinal inflammation has not been reported in HCV chronically-infected patients and, for the vast majority of these patients, no sign of hepatic inflammation can be detected despite the constant and unquestionable viral production in this organ.

Regulatory mechanisms that are induced or exploited by chronic infectious agents may be viewed as an additional way to balance the host-pathogen interaction whereby host tissue damage is restricted and pathogen survival is favored. It is tempting to speculate that interference with TLR4 may be a way in which a specific form of circulating HCV particles contributes to chronicity while preventing infected organs from destructive inflammation and immunity.
